# Response Strategies for Emerging Infectious Diseases: More Efforts Are Needed

**DOI:** 10.3390/tropicalmed8080404

**Published:** 2023-08-08

**Authors:** Yuhao Lin, Tianmu Chen

**Affiliations:** 1 State Key Laboratory of Vaccines for Infectious Diseases, Xiang An Biomedicine Laboratory, School of Public Health, Xiamen University, Xiamen 361102, China; 2 State Key Laboratory of Molecular Vaccinology and Molecular Diagnostics, National Innovation Platform for Industry-Education Integration in Vaccine Research, Xiamen University, Xiamen 361102, China

In recent years, emerging infectious disease outbreaks have placed significant health and socioeconomic burdens upon the population. For example, the COVID-19 epidemic has not only caused over 700 million cumulative cases worldwide, including 6 million cumulative deaths by 18 May 2023 [1], but also cost the global economy over USD 280 billion within its first quarter [2]. Monkeypox cases rapidly increased in several countries in 2022, including the United States, China, Japan, the United Kingdom and so on, seriously affecting the health of the population and disrupting normal economic activities [3]. In addition, epidemics such as AIDS, Ebola and MERS have caused tremendous physical suffering, as well as serious socioeconomic impacts.

Several studies [4,5,6,7] have shown that pharmaceutical interventions (PIs) and non-pharmaceutical interventions (NPIs), such as vaccination, mask-wearing and mandatory isolation, play a significant role in controlling the spread of infectious diseases. They can achieve several objectives, including reducing the number of infections and shortening the duration of the epidemic. However, several challenges remain regarding the study of response strategies for emerging infectious diseases. For example, when there is an outbreak of emerging infectious disease and the epidemiological characteristics of the disease are unclear, many measures proposed by the Center for Disease Control and Prevention (CDC) would be ineffective. As a result, model analysis has gained importance in recent years, as it allows us to predict disease trends, optimize current strategies and assess the effectiveness of comprehensive interventions.

In this Special Issue, we focus on the optimization of response strategies and preventive and control measures used to limit the spread of emerging infectious diseases, thus improving public health prevention and control. A total of five articles feature in this Special Issue.

Three articles refer to compartment models. Xiang et al. explored the optimal varicella vaccination schedule in Jiangsu Province, China [8]. This study shows that it is necessary to promote two-dose varicella vaccination, and the optimal age of patients upon receiving the second dose vaccination is 5–10 years old. Tanatorn et al. assessed the characteristics of COVID-19 transmission in Thailand [9]. Using trade-off analysis, this study indicates that vaccine efficacy is essential to controlling the COVID-19 epidemic. Lou et al. retrospectively explored the control measures implemented in Shanghai to limit the spread of the Omicron variant of COVID-19 [10]. Counterfactual assessment of this study suggests that if Shanghai wants to achieve dynamic-zero status, citywide static management should be implemented as early as possible. The article by Wang et al. applied a multi-agent model. They proposed a strategy formulation framework (SFF) to improve screening efficiency based on the trajectory network [11]. This study reveals that when the first confirmed case appears, it is important to pay more attention to people who have a greater number and frequency of contacts, while during regular screening, CDCs should focus on individuals who always have multiple contacts. Another article developed a screening tool to detect Chikungunya virus (CHIKV) [12]. The advantage of this screening tool is that it can help primary care physicians to deal with common arboviral infections using only clinical symptoms. At the same time, it has high positive predictive value and accuracy, as well as a high Youden index.

These articles used different methodologies (e.g., compartment model analysis, multi-agent model analysis, case series analysis, etc.) from different perspectives (e.g., facilitating clinical diagnosis, improving screening efficiency, controlling an epidemic, etc.) to propose new prevention and control measures to control emerging infectious diseases. Thanks to the efforts of researchers, we can make progress in combating emerging infectious diseases. We invite additional researchers to publish their work in this Special Issue, which is titled “Response Strategies for Emerging Infectious Diseases”.

## References

[1] World Health Organization (2023). COVID-19 Weekly Epidemiological Update, Edition 143, 18 May 2023.

[2] Naseer S., Khalid S., Parveen S., Abbass K., Song H., Achim M.V. (2022). COVID-19 outbreak: Impact on global economy. Front. Public Health.

[3] Saied A.A., Metwally A.A., Choudhary O.P. (2022). Monkeypox: An extra burden on global health. Int. J. Surg..

[4] Dashtbali M., Mirzaie M. (2021). A compartmental model that predicts the effect of social distancing and vaccination on controlling COVID-19. Sci. Rep..

[5] Bouchnita A., Chekroun A., Jebrane A. (2020). Mathematical Modeling Predicts That Strict Social Distancing Measures Would Be Needed to Shorten the Duration of Waves of COVID-19 Infections in Vietnam. Front. Public Health.

[6] Lopes P.H., Wellacott L., de Almeida L., Villavicencio L.M.M., Moreira A.L.L., Andrade D.S., Souza A.M.C., de Sousa R.K.R., Silva P.S., Lima L. (2022). Measuring the impact of nonpharmaceutical interventions on the SARS-CoV-2 pandemic at a city level: An agent-based computational modelling study of the City of Natal. PLoS Glob. Public Health.

[7] Rafiei H., Salehi A., Baghbani F., Parsa P., Akbarzadeh T.M. (2023). Interval type-2 Fuzzy control and stochastic modeling of COVID-19 spread based on vaccination and social distancing rates. Comput. Methods Programs Biomed..

[8] Sun X., Dai C., Wang K., Liu Y., Jin X., Wang C., Yin Y., Ding Z., Lu Z., Wang W. (2022). A Dynamic Compartmental Model to Explore the Optimal Strategy of Varicella Vaccination: An Epidemiological Study in Jiangsu Province, China. Trop. Med. Infect. Dis..

[9] Intarapanya T., Suratanee A., Pattaradilokrat S., Plaimas K. (2023). Modeling the Spread of COVID-19 with the Control of Mixed Vaccine Types during the Pandemic in Thailand. Trop. Med. Infect. Dis..

[10] Lou L., Zhang L., Guan J., Ning X., Nie M., Wei Y., Chen F. (2023). Retrospective Modeling of the Omicron Epidemic in Shanghai, China: Exploring the Timing and Performance of Control Measures. Trop. Med. Infect. Dis..

[11] Wang S., Zhang Y., Zhang Q., Lu Q., Liu C., Yi F. (2023). A Strategy Formulation Framework for Efficient Screening during the Early Stage of a Pandemic. Trop. Med. Infect. Dis..

[12] Rueda J.C., Pelaez-Ballestas I., Angarita J.I., Santos A.M., Pinzon C., Saldarriaga E.L., Rueda J.M., Forero E., Saaibi D.L., Pavia P.X. (2023). Clinical Diagnosis of Chikungunya Infection: An Essential Aid in a Primary Care Setting Where Serological Confirmation Is Not Available. Trop. Med. Infect. Dis..

